# Disease spectrum and its molecular characterisation in the lentil production system of lower-Indo Gangetic plains

**DOI:** 10.3389/fpls.2024.1199016

**Published:** 2024-02-23

**Authors:** Huma Nazneen, Raju Das, Arpita Das, Subrata Dutta, Sudip Bhattacharya, Subhas Patar, Subhadeep Roy, Sanjeev Gupta, Shiv Kumar

**Affiliations:** ^1^ Bidhan Chandra Krishi Viswavidyalaya, Mohanpur, West Bengal, India; ^2^ Division of Crop Science, Indian Council of Agricultural Research, Krishi Bhawan, New Delhi, India; ^3^ South Asia & China Regional Programme, International Centre for Agricultural Research in the Dry Areas (ICARDA), New Delhi, India

**Keywords:** lentil, collar rot, blight complex, rust, molecular characterization, Geospatial mapping

## Abstract

Lentil is a food legume grown in the Indo-Gangetic plains including lower Gangetic Bengal (LGB). Lentil productivity in this zone is severely impeded because of the prevalence of several biotic cues. Plausible reports regarding the status of disease scenario and the associated risk factors are missing. Therefore, judicious crop management strategies are lacking. An intensive survey of 267 farmers’ fields was conducted over 3 years in major lentil-growing districts of LGB to evaluate the disease incidence and prevalence. Additional insights were generated, apprehending isolation and characterisation of associated pathogens through spore morphology and molecular markers as well as elucidating the role of biophysical factors in influencing disease development. Climate change has shifted the disease dimension of lentil and precipitated new disease complexes of great risk, which was reflected through geospatial mapping results in the present study. The prevalence of three major diseases, namely collar rot (*Sclerotium rolfsii*), lentil blight complex (LBC) incited by both *Alternaria* and *Stemphylium*, and lentil rust (*Uromyces viciae-fabae*), was ascertained through cultural and molecular studies and contextualised through pathogenicity appraisal. This study is the first to investigate the complex mixed infection of *Alternaria alternata* and *Stemphylium botryosum*, successfully isolating *S. botyrosum* in India, and confirming the pathogens through sequencing by using internal transcribed spacer (ITS) primers and *Stemphylium*-specific Glycerol-3-phosphate dehydrogenase 1 (gpd1) and gpd2 primers. Unlike late planting, early planting promoted collar rot infestation. LBC and rust incidence were magnified in late planting. Soil texture resulted in the spatial distribution of collar rot disease. The surveyed data also highlighted the potential role of resistant cultivars and cropping pattern intervention to ensure associational resistance towards addressing the disease bottleneck in lentil.

## 1 Introduction

Lentil (*Lens culinaris* Medik.) is one of the world’s most predominant cool-season pulse crops. Globally, this crop covers approximately 5.01 Mha area and has a production of 6.54 MMT. Canada is the largest producer of lentil in the world, followed by India (1.46 Mha). India is also the highest consumer of lentil (FAOSTAT, 2021). In 2020, India produced 1.18 MMT of lentil, which was grown in Indo-Gangetic plains (IGP) and Central India. Thus, these areas all together accounted for 80%–90% of the total area under lentil production in India (FAOSTAT, 2021). This food legume is a crucial component of cereal-based cropping system of IGP (Bhattacharya et al., 2022a, Bhattacharya et al., 2022b). However, the average productivity of lentil in IGP is low due to several biotic constraints and farmers being sceptical of converting the traditional rice-based monocropping system to a more productive rice–lentil cropping system. Moreover, climate change in this region is influencing the dynamics of biotic and abiotic cues that severely impede lentil productivity. Earlier reports have revealed that collar rot, *Stemphylium* and *Alternaria* blight and rust are major biotic vagaries that decelerate lentil cultivation in this region and other South Asian countries (Kushwaha et al., 2019; Mondal et al., 2021; Roy et al., 2021; Singh et al., 2021).

In IGP, lentil is traditionally planted in a standing rice crop 7–10 days before harvest as a paira crop (relay cropping). However, this triggers congenial conditions for an array of pathogenic invasions (Maji et al., 2019). Collar rot is the most detrimental soil-borne fungal disease of rice–fallow niches that develop at the initial crop growth stage, whereas, *Alternaria* and *Stemphylium* blight as well as rust (*Uromyces viciae-fabae*) during the flowering stage are devastating diseases. The symptoms of *Alternaria* blight are mostly indistinguishable from those of *Stemphylium* blight as the causative pathogens in both these diseases attack lentil plants simultaneously in a complex form during pre-flowering and flowering stages and hinder their proper management (Das et al., 2019). The sub-tropical humid regions of IGP have a moderate temperature and high humidity, this disease complex is spreading extensively, leading to complete defoliation and subsequent yield losses of up to 80% because most lentil cultivars are susceptible to both diseases (Salam et al., 2016). The complex nature of the lentil blight disease warrants the need to explicitly identify the pathogens at cultural and molecular levels.

Lack of information regarding the impact of resistant varieties in farmers’ fields *vis a vis* the prevalence of major diseases in lentil-growing areas needs to be examined in a holistic manner. Geostatistical approaches have been frequently used to describe plant disease patterns geographically and to identify the potential risk factors for epidemics (Byamukama et al., 2014). Additionally, a geographic information system can be used to characterise the pathogen’s spatial locations and identify the disease-affected areas, thereby ensuring judicious crop management (Guo, 2018). Thus, to minimise the potential risk associated with lentil cultivation, immediate interventions of scientific communities are required for livelihood security and nutritional sustenance of poor farmers. The present study integrates three complementary objectives to (i) assess the incidence, severity, and prevalence of major fungal diseases of lentil; (ii) characterise the disease-associated pathogens at cultural and molecular levels; and (iii) determine biophysical factors, such as soil texture, varietal effect, intercropping patterns, and sowing periods, that are associated with disease prevalence in lentil by conducting surveys and surveillance in old and new alluvial zones of Gangetic Bengal (henceforth lower Gangetic Bengal; LGB).

## 2 Materials and methods

### 2.1 Survey sites and weather data

**Figure 1:**
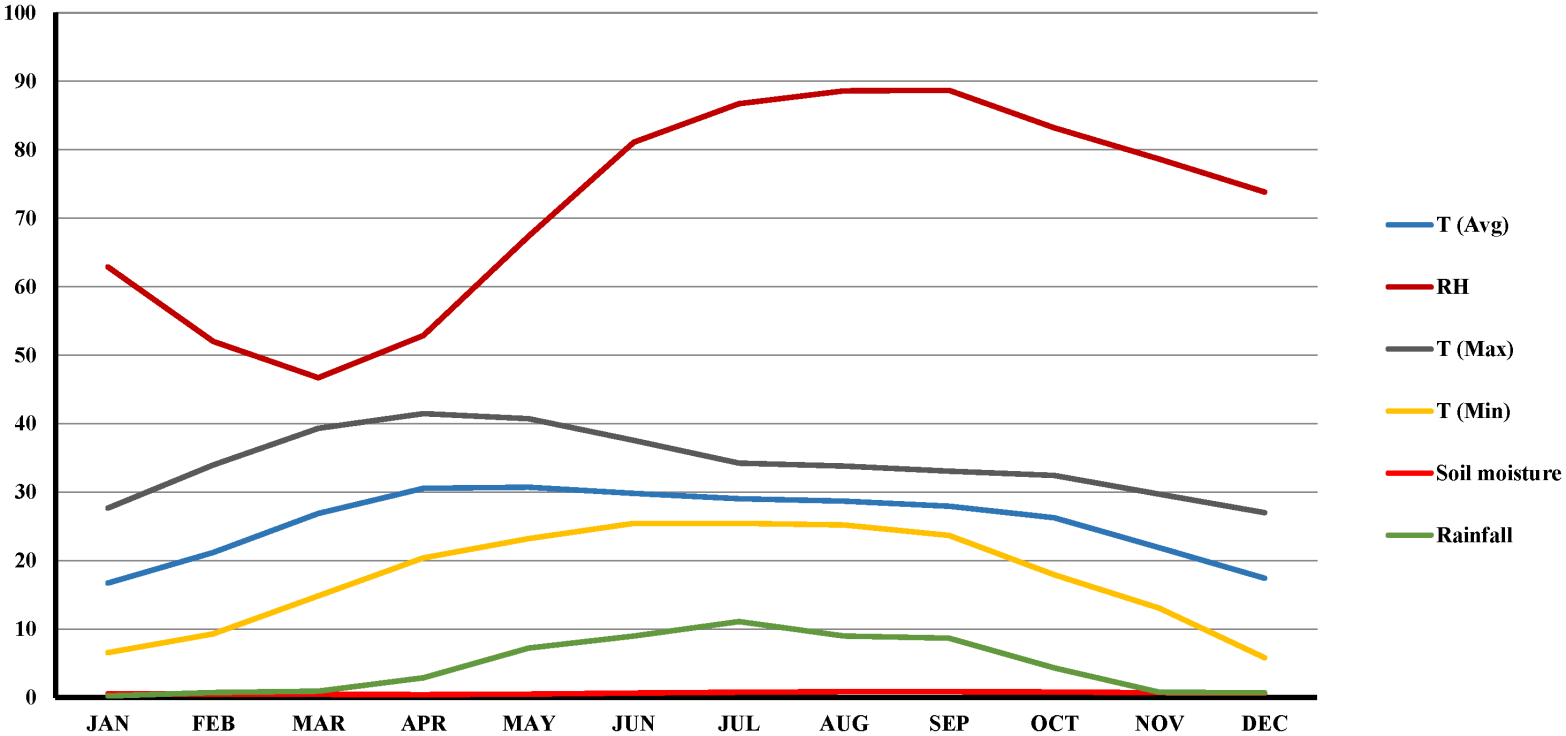
Average temperature, relative humidity and rainfall during lentil growing periods (standards weeks) across the surveyed locations over three consecutive years (2018-19, 2019-20 and 2020-2021).

An intensive survey was conducted in major lentil growing districts of LGB, *viz*., North 24 Parganas (latitude of 22.61°N; longitude of 88.40°E with 7 m above mean sea level), Nadia (latitude of 23.47°N; longitude of 88.55°E with 12 m above mean sea level [AMSL]), Murshidabad (latitude of 24.17°N; longitude of 88.28°E with 10 m AMSL), and Malda (latitude of 25.01°N; longitude of 88.14°E with 17 m AMSL) over three consecutive years (2018-19, 2019-20, and 2020-21) during the months of November–March (**Supplementary Table S1**). A geographical positioning system (GPS) was used to record the latitude, longitude, and location of each surveyed field. The surveyed districts were decisively chosen considering their maximum lentil acreage (74.5%) (Anonymous, 2020-2021). For disease evaluation and sample collection, 267 fields were assessed, and parameters such as cropping patterns, sowing time, field size, latitude, and longitude were recorded (**Supplementary Table S1**). The field size of the surveyed areas ranged from 0.14 ha to 0.40 ha. Most of the soil in the surveyed fields was sandy clay loam to clay loam in nature. The climate of the surveyed districts was characterized by sub-tropical monsoons. The temperature during the winter season varied from 6 to 32°C, with an average temperature of 19°C, relative humidity (RH) of 70.42%, and average rainfall of 2.01 mm during the entire crop growing season over the three consecutive years (**Figure 1**).

### 2.2 Sampling methodology

Along an “X”-shaped transect throughout the field, plants were examined for disease incidence and severity. A 50 × 50 m^2^ polygon within the field was delimited using the GPS and selected as the sampling region. The first point was not less than 10 m from the edge of the field, with the rest of the points each approximately 6 m apart. Fifteen plants were sampled along an “X” transect from each field. Out of these 15 plants, a minimum of 3 and a maximum of 5 diseased plants/parts (leaf samples for blight and rust, whereas root samples for collar rot), depending upon the disease incidence, were selected for further confirmation through isolation and subsequent culture. For collar rot, the roots of five plants were longitudinally slit and checked for vascular discoloration, and soil samples were also collected for confirmation of the presence of sclerotia. The estimation of sclerotial population in the soil for the assessment of collar rot was performed through the rapid flotation-sieving technique (Rodriguez-Kabana et al., 1974). Soil texture estimation was done through the hydrometer method (Gee and Bauder, 1979). Subsequently, a DBF file consisting of X and Y coordinate data with respect to sampling site location was created. A shape file (vector data) showing the outline of the LGB area was created in Arc GIS v. 10.3 software. The DBF file was opened in the project window, and in the X-field longitudes and in the Y-field latitudes were selected. The Z field was used for disease data and sclerotia population with respect to the type of map to be generated. Different lentil disease maps were generated using Arc GIS v. 10.3, employing krigging as the interpolation method as it provides the best linear unbiased estimates (Mueller, 2004). The generated map was reclassified based on ratings for the respective diseases.

### 2.3 Disease assessment

In most of the surveyed sites, three diseases were common. During the early seedling stage, there was collar rot infestation followed by lentil blight in the form of a complex (henceforth lentil blight complex: LBC) due to mixed infection of both *Alternaria* and *Stemphylium* as well as rust. In accordance with Madden et al. (2008), disease incidence, severity, and prevalence were deployed to appraise the disease distribution and damage. Disease prevalence (DP) was estimated as the percentage of fields where the disease was detected (Nutter et al., 1991). It was estimated as the percentage ratio of an area’s infected fields to its sampled fields in a district. Plants were evaluated for collar rot using an ordinal disease rating scale (0-9) based on root rot symptoms and lower stem vascular discoloration, where 0=immune (No mortality); 1=highly resistant (less than 1% mortality); 3=resistant (1-10% mortality); 5=moderately susceptible (11-20% mortality); 7=susceptible (21-50% mortality); 9=highly susceptible (51% or more mortality) (Hussain et al., 2005). The percent disease incidence (PDI) was calculated using the formula devised by Mathur et al. (1972). Disease incidence (DI) for collar rot was determined as the number of plants infected, expressed as a percentage of the total number of units assessed per field. The average DI for each pathogen was obtained as the sum of the DI values for each field divided by the number of fields surveyed, expressed as a percentage.


$$\text{Percent disease incidence }=\;\frac{\text{Total infected plant}}{\text{Total emergence of plant}}\times 100$$

**Figure 2:**
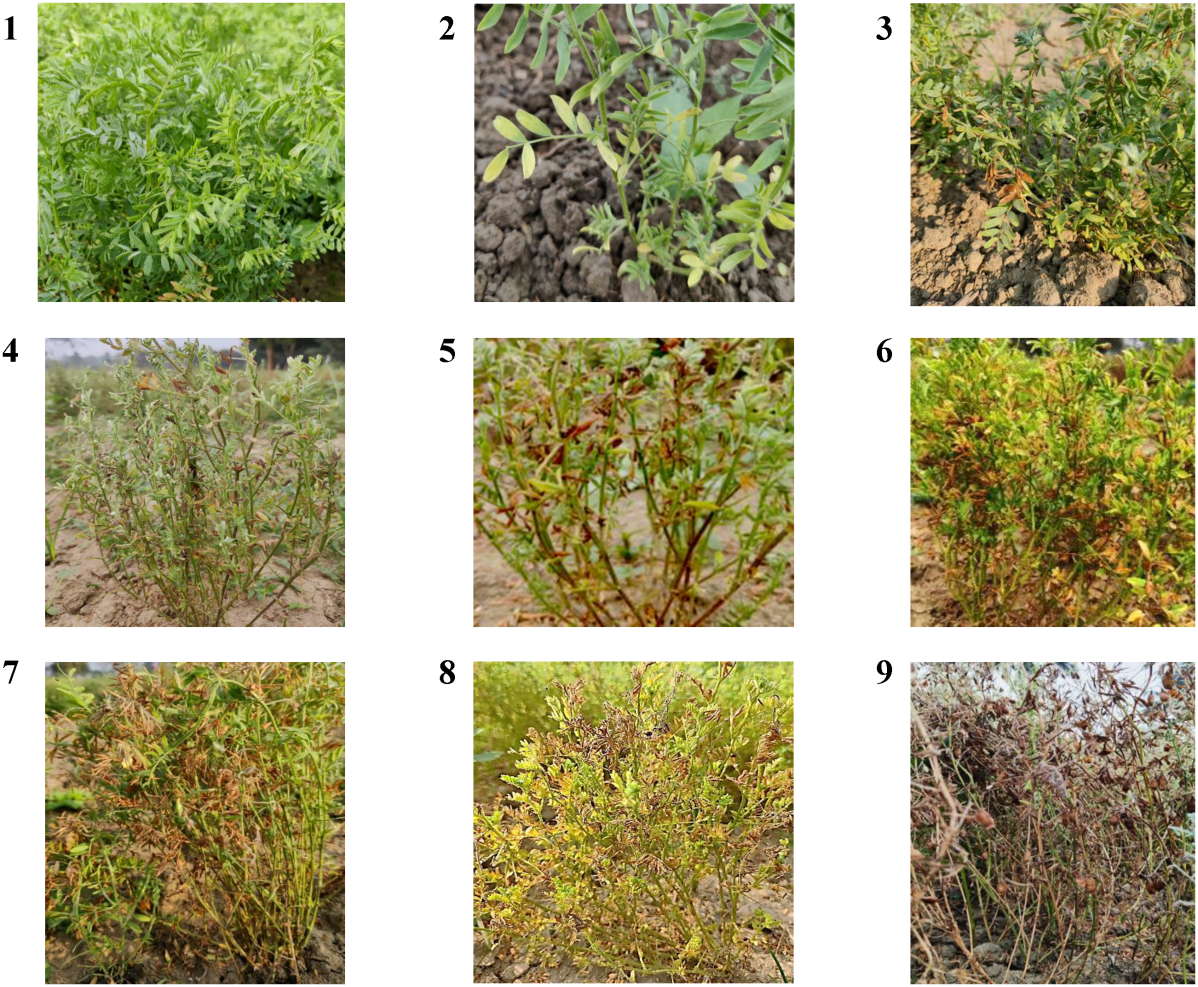
Image based observation of 1-9 disease grading scale of Lentil blight complex (LBC) disease.


Disease severity (DS) of LBC and rust was evaluated as the percentage diseased area in the sampling unit (plant, leaf, etc.). Each pathogen was assigned a disease rating scale and severity grade. Severity was rated on lentil plants and leaves from 15 plants along the transect in each field, and plants were assessed and rated for LBC using a disease rating scale modified from Kant et al. (2017) based on blight symptoms, where 1=no infection (resistant: R); 2=few chlorotic lesion (R to moderately resistant: MR); 3=expanding lesion on 6 to 15% leaves turning necrotic (MR); 4 = 16 to 30% leaves discoloured (MR to moderately susceptible: MS); 5 = 31 to 45% leaves discoloured (MS); 6 = 46 to 60% leaves discoloured and defoliated (MS to susceptible: S); 7 = 61 to 80% leaves discoloured and defoliated (S); 8 = 81 to 90% leaves discoloured, symptom on stem (S to very susceptible: VS); 9 = whole plant death (VS) (**Figure 2**). For rust disease, lentil plants were assessed and rated using a disease rating scale (1-9) modified from Khare et al. (1993), where 1=no pustules visible (highly Resistant); 3=few scattered pustules, usually seen after careful searching (resistant); 5=pustules common on leaves and easily observed but causing no apparent damage (moderately resistant); 7=pustule are very common and damaging, with few pustules on petioles and stems (susceptible); 9=pustules are very extensive on all parts, with some death of leaves and other plant parts (highly susceptible). Disease severity scores were converted into a percent severity index (PSI) for foliar diseases like LBC and rust and calculated using the following formula (Wheeler, 1969):

$$\text{Percent disease index }=\;\frac{\text{Sum of numerical ratings}}{\text{Total number of observations}}\;\times \;\frac{100}{\text{Maximum disease score}}$$

### 2.4 Isolations, identification, and pathogenicity tests

Root pieces with disease symptoms were inoculated on potato dextrose agar (PDA, Himedia, India) (Rangaswami and Mahadevan, 1999) and incubated at 27 ± 2°C for 7 to 10 days for identification of the collar rot pathogen. Accordingly, leaf samples were collected for the detection of LBC caused by a mixed infection of *Alternaria* and *Stemphylium* and through microscopy, which confirmed the presence of both pathogens. These pathogens were isolated by single spore microscopy, inoculated on PDA (by using dextrose in half quantity), and incubated at 24 ± 1°C for nearly two weeks. The fragments of hyphal growth from the growing tips in this culture, were then transferred to a fresh PDA culture. All the detected fungi were purified and kept at 4°C on PDA slants. Similarly, the prevalence of rust disease in the fields was affirmed through microscopic confirmation of uredospore from the collected leaf samples. For each disease observed in the field, 7 - 10 samples per disease materials were cultured. To establish Koch’s postulates, one representative isolate from each disease was used to inoculate lentil plants. All the cultures obtained from the infected areas of the plant were tested for their respective pathogens *viz*., *S. rolfsii*, *A. alternata*, and *S. botryosum*. By performing pathogenicity assays on susceptible lentil cv. HUL-57 for *S. rolfsii* and RKL-22-10 for *A. alternata* and *S. botryosum*, all fungal species were tested for confirmation. Seeds were rinsed with autoclaved double-distilled water after being sterilised with 1% sodium hypochlorite solution, and then plants were inoculated with 10-15 sclerotia/pot for testing collar rot and 5×10^4^ conidia ml^-1^ of LBC for re-isolation of the pathogens.

### 2.5 Fungal DNA isolation, amplification and sequencing

For molecular characterization, mycelia of freshly grown fungal cultures (*S. rolfsii*, *S. botryosum* and *A. alternata*) in potato dextrose broth (PDB) were harvested and crushed into a fine powder with liquid nitrogen. Accordingly, rust DNA (*U. fabae*) was extracted directly from the diseased leaf samples. Total genomic DNA of the different fungal cultures was isolated directly using the HiGenoMB HiPurA fungal DNA purification kit (Himedia, India), according to the manufacturer’s protocol. Gene fragments, internal spacer rDNA region (ITS) universal primer pair ITS 1 (CTT GGT CAT TTA GAG GAA GTA A) and ITS 4 (TCC TCC GCT TAT TGA TAT GC) (White et al., 1990) were used for PCR amplification for all the pathogens (*S. rolfsii*, *S. botryosum*, *A. alternata*, and *U. fabae*) and glyceraldehyde-3-phosphate dehydrogenase (GAPDH) specific primers gpd1 (CAA CGG CTT CGG TCG CAT TG) and gpd2 (GCC AAG CAG TTG GTT GTG C) for amplification of *S. botryosum* and *A. alternata* (Berbee et al., 1999). A 25 μl PCR reaction mixture was applied, comprising of 3 μl of PCR buffer (Thermo Fisher Scientific, India), 0.2 μl of Taq polymerase (Thermo Fisher Scientific, India), 1 μl of dNTP’s, 1 μl of each primer, 1 μl of template DNA, and 17.8 μl of molecular biology grade water. The PCR programme was: initial denaturation of 1 cycle at 94°C for 2 min; denaturation of 35 cycles at 94°C for 4 min; annealing at 45°- 60°C for 1 min; extension at 72°C for 1 min; and final extension of 1 cycle at 72°C for 5 min; and then held at 4°C. The obtained sequences were searched for homologous sequences from NCBI database by performing BLASTN (http://www.ncbi.nlm.nih.gov/BLAST/) analysis. The homologous sequences obtained from NCBI GenBank were aligned using multiple sequence alignment, and alignment of approximately 500 bp sequences was performed using CLUSTALW version 1.8 (https://www.genome.jp/tools-bin/clustalw). A phylogenetic tree was constructed with the evolutionary distances using the neighbour joining method (Saitou and Nei, 1987). Tree topologies were evaluated by performing bootstrap analysis of 1,000 datasets (Felsenstein, 1985) with the MEGA 4 package (Tamura et al., 2007).

## 3 Results

### 3.1 Fungal disease incidence in surveyed sites

In the surveyed LGB districts, lentil was majorly cultivated as a sole crop (75.03%), whereas intercropping was practised for 24.97% of lentil (**Supplementary Table S1**). In most locations, farmers preferred to grow lentil in the rice–fallow system or intercrop it with rice/vegetables such as cucurbits or okra/mustard/maize, or forest trees such as lambu (African mahogany: *Khaya senegalensis*)/shisham (*Dalbergia sissoo*). Normal sowing of lentil was mostly performed during November, whereas late sowing was performed in December. Amid the surveyed sites, 40% fields were normally sown, while 60% fields were late sown because of the late harvesting of the preceding rice crop. The survey reflected that the lentil fields majorly developed three fungal diseases, namely collar rot, LBC, and rust. Collar rot and LBC were the most widespread diseases, whereas the intensity of lentil rust was less.

In each cropping season, different districts recorded varied levels of disease parameters (DP, DI, and DS) (**Tables 1**, **2**). The mean DP was the highest for LBC, followed by collar rot and rust. The mean DI for collar rot was the highest in the third year of survey (2020–2021), whereas the highest DS was observed for LBC and rust during the first (2018–2019) and second year (2019–2020), respectively. In total, 81 lentil fields were surveyed during the first year (2018–2019). The maximum mean DP was recorded for collar rot (91.21%), followed by LBC (88.26%). DS was the highest for LBC (11.96% ± 0.77%). During the second-year survey (2019–2020), 97 fields were covered. The maximum mean DP was recorded for LBC (96.03%), followed by rust (90.49%). Collar rot was less prevalent, whereas drastic emergence of LBC and rust diseases were observed compared with the previous year. DS was the highest for rust disease (15.45% ± 0.69%). During the third year of survey (2020–2021), 89 fields were covered. All three fungal diseases were more severe than those in both previous years. The maximum mean DP (100%) and DS were observed for LBC (11.88% ± 0.38%) (**Table 1**).

**Table 1:** Disease Prevalence (DP) in lentil fields across the surveyed districts of LGB during three years (2018-2021).

Lower Gangetic Bengal		Collar rot				Blight complex				Rust			
Districts	Blocks	2018-19	2019-20	2020-21	Mean	2018-19	2019-20	2020-21	Mean	2018-19	2019-20	2020-21	Mean
**Nadia**	Chakdaha	100.00	92.31	87.50	93.27	83.33	100.00	100.00	94.44	83.33	100.00	100.00	94.44
	Ranaghat	83.33	50.00	100.00	77.78	83.33	100.00	100.00	94.44	83.33	100.00	100.00	94.44
	C-Block (Kalyani)	83.33	100.00	75.00	86.11	83.33	100.00	100.00	94.44	0.00	100.00	100.00	66.67
	Krishnagar-I	100.00	100.00	100.00	100.00	100.00	100.00	100.00	100.00	83.33	100.00	100.00	94.44
	Chapra	83.33	83.33	100.00	88.89	100.00	100.00	100.00	100.00	100.00	100.00	75.00	91.67
	Karimpur-I	87.50	75.00	100.00	87.50	100.00	100.00	100.00	100.00	87.50	100.00	75.00	87.50
		**89.58**	**83.44**	**93.75**	**88.92**	**91.67**	**100.00**	**100.00**	**97.22**	**72.92**	**100.00**	**91.67**	**88.19**
**Murshidabad**	Sagardighi	87.50	82.35	100.00	89.95	87.50	94.12	100.00	93.87	37.50	88.24	94.74	73.49
	Jiaganj	100.00	72.73	100.00	90.91	75.00	100.00	100.00	91.67	75.00	72.73	100.00	82.58
	Berhampore	100.00	100.00	100.00	100.00	100.00	100.00	100.00	100.00	100.00	100.00	100.00	100.00
		**95.83**	**85.00**	**100.00**	**93.62**	**87.50**	**98.04**	**100.00**	**95.18**	**70.83**	**86.99**	**98.25**	**85.36**
**Malda**	English Bazar	83.33	75.00	100.00	86.11	66.67	100.00	100.00	88.89	0.00	75.00	75.00	50.00
	Kaliachak-2	100.00	100.00	75.00	91.67	100.00	100.00	100.00	100.00	100.00	75.00	100.00	91.67
	Gazal block	80.00	83.33	100.00	87.78	80.00	83.33	100.00	87.78	80.00	100.00	100.00	93.33
		**87.78**	**86.00**	**91.67**	**88.52**	**82.22**	**94.44**	**100.00**	**92.22**	**60.00**	**83.33**	**91.67**	**78.33**
**North 24 Parganas**	Habra -1	75.00	50.00	100.00	75.00	100.00	100.00	100.00	100.00	75.00	100.00	100.00	91.67
	Barasat block-1	100.00	100.00	75.00	91.67	100.00	75.00	100.00	91.67	100.00	75.00	100.00	91.67
	Barasat block-2	100.00	75.00	100.00	91.67	75.00	100.00	100.00	91.67	75.00	100.00	57.14	77.38
**Mean**		**91.67**	**75.00**	**91.67**	**86.11**	**91.67**	**91.67**	**100.00**	**94.44**	**83.33**	**91.67**	**85.71**	**86.90**
**Grand Mean**		**91.21**	**82.36**	**94.27**	**89.29**	**88.26**	**96.03**	**100**	**94.77**	**71.77**	**90.49**	**91.82**	**84.70**

Bold values refers to the average incidence and severity of respective districts and overall average.

**Table 2:** Disease incidence (DI) of collar rot and Disease severity (DS) of blight complex and rust in lentil fields across the surveyed districts of LGB during three years (2018-2021).

Lower Gangetic Bengal		Collar rot				Blight complex				Rust			
Districts	Blocks	2018-19	2019-20	2020-21	Mean	2018-19	2019-20	2020-21	Mean	2018-19	2019-20	2020-21	Mean
**Nadia**	Chakdaha	2.50± 0.56	6.85 ± 1.14	6.88 ± 1.29	5.89 ± 0.75	21.55 ± 4.71	16.24 ± 1.94	13.11 ± 0.92	16.49 ± 1.48	25.67 ± 6.54	22.60 ± 1.44	12.81 ± 0.61	20.38 ± 1.82
	Ranaghat	5.00± 2.03	2.50 ± 1.89	4.50 ± 0.65	4.14 ± 1.02	10.74 ± 4.50	16.74 ± 2.18	13.18 ± 0.39	13.15 ± 2.03	19.10 ± 4.79	18.87 ± 1.70	12.35 ± 1.41	17.11 ± 2.19
	C-Block (Kalyani)	14.50 ± 3.40	15.25 ± 2.02	15.75 ± 5.50	15.07 ± 2.05	7.12 ± 1.50	18.54 ± 0.32	12.54 ± 0.57	11.93 ± 1.46	0.00 ± 0.00	18.94 ± 0.98	13.81 ± 0.98	9.36 ± 2.34
	Krishnagar-I	7.83± 2.60	10.25 ± 1.49	17.40 ± 3.97	11.67 ± 1.96	26.14 ± 4.31	13.65 ± 0.32	17.14 ± 0.78	19.81 ± 2.18	19.43 ± 4.10	19.68 ± 0.70	15.85 ± 0.71	18.30 ± 1.64
	Chapra	5.17± 1.38	4.00 ± 1.55	6.25 ± 1.11	5.00 ± 0.81	29.17 ± 1.48	17.45 ± 1.35	18.71 ± 1.91	22.16 ± 1.63	23.38 ± 3.37	19.65 ± 1.90	17.30 ± 5.79	20.46 ± 1.99
	Karimpur-I	8.50± 4.19	8.00 ± 2.71	12.25 ± 0.85	9.31 ± 2.17	20.69 ± 3.26	19.69 ± 1.46	13.73 ± 1.19	18.70 ± 1.79	17.78 ± 2.94	20.47 ± 3.42	13.44 ± 4.50	17.37 ± 2.01
		**7.32 ± 1.26**	**7.34 ± 0.88**	**10.24 ± 1.35**	**8.16 ± 1.10**	**19.31 ± 1.85**	**16.86 ± 0.83**	**14.60 ± 0.58**	**17.13 ± 0.78**	**17.57 ± 2.03**	**20.67 ± 0.77**	**14.12 ± 0.99**	**17.65 ± 0.88**
**Murshidabad**	Sagardighi	5.00 ± 1.27	10.24 ± 1.61	10.74 ± 0.93	9.50 ± 0.83	11.81 ± 3.22	12.14 ± 0.60	10.21 ± 0.60	11.25 ± 0.74	8.61 ± 4.33	15.12 ± 1.66	9.44 ± 0.67	11.49 ± 1.11
	Jiaganj	2.50 ± 0.65	6.55 ± 2.27	11.60 ± 2.14	7.00 ± 1.50	4.72 ± 1.72	11.51 ± 0.35	15.23 ± 1.45	11.08 ± 0.95	11.04 ± 4.17	10.57 ± 2.18	8.77 ± 0.40	10.21 ± 1.40
	Berhampore	20.50 ± 3.10	10.75 ± 1.89	18.00 ± 5.10	16.42 ± 2.26	32.33 ± 2.64	13.54 ± 1.14	15.45 ± 0.86	20.44 ± 2.70	12.29 ± 1.14	13.09 ± 1.05	13.18 ± 0.77	12.86 ± 0.54
		**8.25 ± 2.07**	**9.03 ± 1.20**	**11.93 ± 1.07**	**9.93 ± 0.78**	**15.17 ± 3.17**	**12.10 ± 0.58**	**11.86 ± 0.67**	**12.66 ± 0.75**	**10.14 ± 2.34**	**13.30 ± 1.20**	**9.86 ± 0.54**	**11.37 ± 0.75**
**Malda**	English Bazar	4.00 ± 1.65	5.25 ± 2.29	5.25 ± 1.65	4.71 ± 1.00	5.08 ± 2.04	6.72 ± 2.01	9.61 ± 1.04	6.84 ± 1.14	0.00 ± 0.00	8.19 ± 3.05	8.00 ± 2.96	4.63 ± 1.56
	Kaliachak-2	3.00 ± 1.22	3.50 ± 1.19	2.50 ± 1.04	3.00 ± 0.62	12.08 ± 2.28	9.41 ± 0.83	9.71 ± 0.76	10.40 ± 0.85	12.29 ± 1.14	10.72 ± 1.14	10.28 ± 0.41	11.10 ± 1.33
	Gazal block	3.00 ± 1.79	5.00 ± 1.88	10.22 ± 2.49	6.85 ± 1.46	7.96 ± 2.00	7.04 ± 1.86	8.42 ± 0.44	7.89 ± 0.73	8.71 ± 2.47	10.00 ± 1.31	5.85 ± 0.59	7.81 ± 0.84
		**3.40 ± 0.89**	**4.64 ± 1.03**	**7.24 ± 1.58**	**5.20 ± 0.75**	**7.91 ± 1.35**	**7.63 ± 0.99**	**9.00 ± 0.38**	**8.23 ± 0.55**	**6.18 ± 1.62**	**9.69 ± 1.45**	**7.40 ± 0.83**	**7.70 ± 0.77**
**North 24 Parganas**	Habra -1	3.75 ± 2.17	4.00 ± 1.89	4.00 ± 0.41	3.50 ± 0.89	11.96 ± 0.77	12.86 ± 1.05	8.84 ± 1.59	11.22 ± 0.81	10.52 ± 3.89	13.49 ± 0.91	9.09 ± 0.86	11.03 ± 1.35
	Barasat block-1	2.50 ± 0.65	3.75 ± 1.44	3.75 ± 1.31	3.33 ± 0.64	9.69 ± 0.78	7.28 ± 2.63	12.00 ± 0.74	9.66 ± 1.04	11.08 ± 0.94	12.51 ± 4.54	16.39 ± 2.35	13.33 ± 1.71
	Barasat block-2	3.00 ± 1.08	5.13 ± 1.78	5.71 ± 1.23	4.89 ± 0.89	8.58 ± 2.89	10.46 ± 0.46	9.43 ± 0.34	9.68 ± 0.61	9.24 ± 3.31	13.73 ± 0.63	7.03 ± 2.55	10.31 ± 1.32
**Mean**		**3.08 ± 0.77**	**4.19 ± 1.04**	**4.73 ± 0.69**	**4.07 ± 0.50**	**10.08 ± 1.00**	**10.26 ± 0.84**	**9.96 ± 0.56**	**10.10 ± 0.46**	**10.28 ± 1.58**	**13.36 ± 1.09**	**10.07 ± 1.66**	**11.35 ± 0.85**
**Grand Mean**		**6.15 ± 0.77**	**6.99 ± 0.58**	**9.27 ± 0.69**	**7.49 ± 0.40**	**15.01 ± 1.21**	**12.87 ± 0.53**	**11.88 ± 0.38**	**13.19 ± 0.44**	**12.91 ± 1.22**	**15.45 ± 0.69**	**10.81 ± 0.55**	**13.13 ± 0.50**

Data is represented as mean ± SE. Disease incidence (DI) of collar rot was determined as the number of plant infected, expressed as a percentage of the total number of units assessed per field. Disease severity (DS) of blight complex and lentil rust was evaluated as the percentage diseased area in the sampling unit (plant, leaf etc.). Severity was rated on lentil plants and leaves from fifteen plants along the transect in each field, using the respective scales for each disease. Disease severity scores were converted into percentage severity index mentioned in the table. Bold values refers to the average incidence and severity of respective districts and overall average.

### 3.2 Association of disease parameters, and cultural and biophysical practices

**Figure 3:**
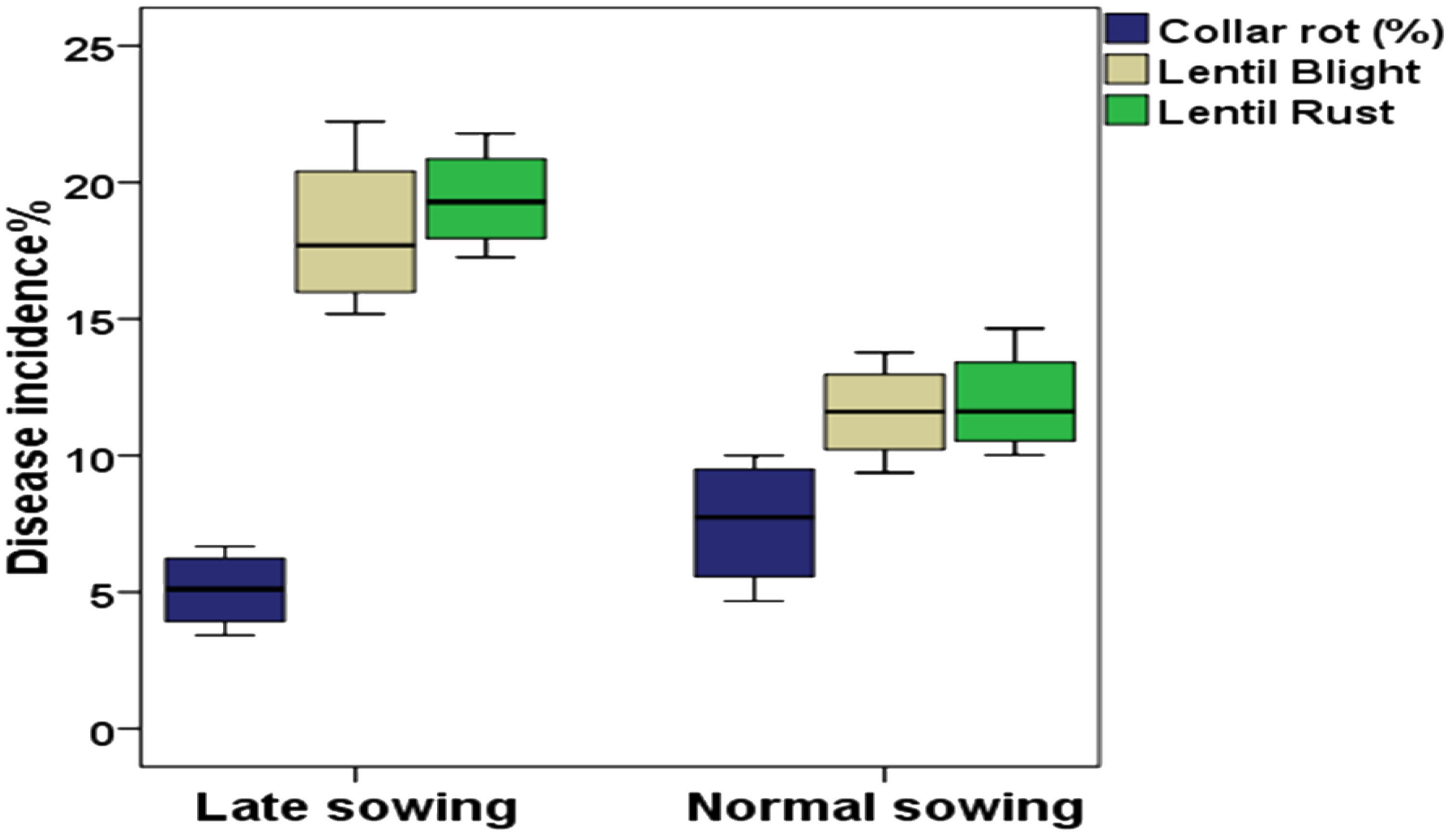
Box plot with different colours representing percentage of disease incidence with different sowing periods. Blue colour – Collar rot disease, Grey colour – Lentil blight complex (LBC); Green colour – Rust disease. The horizontal line in the box indicates the median of the data values.

**Figure 4:**
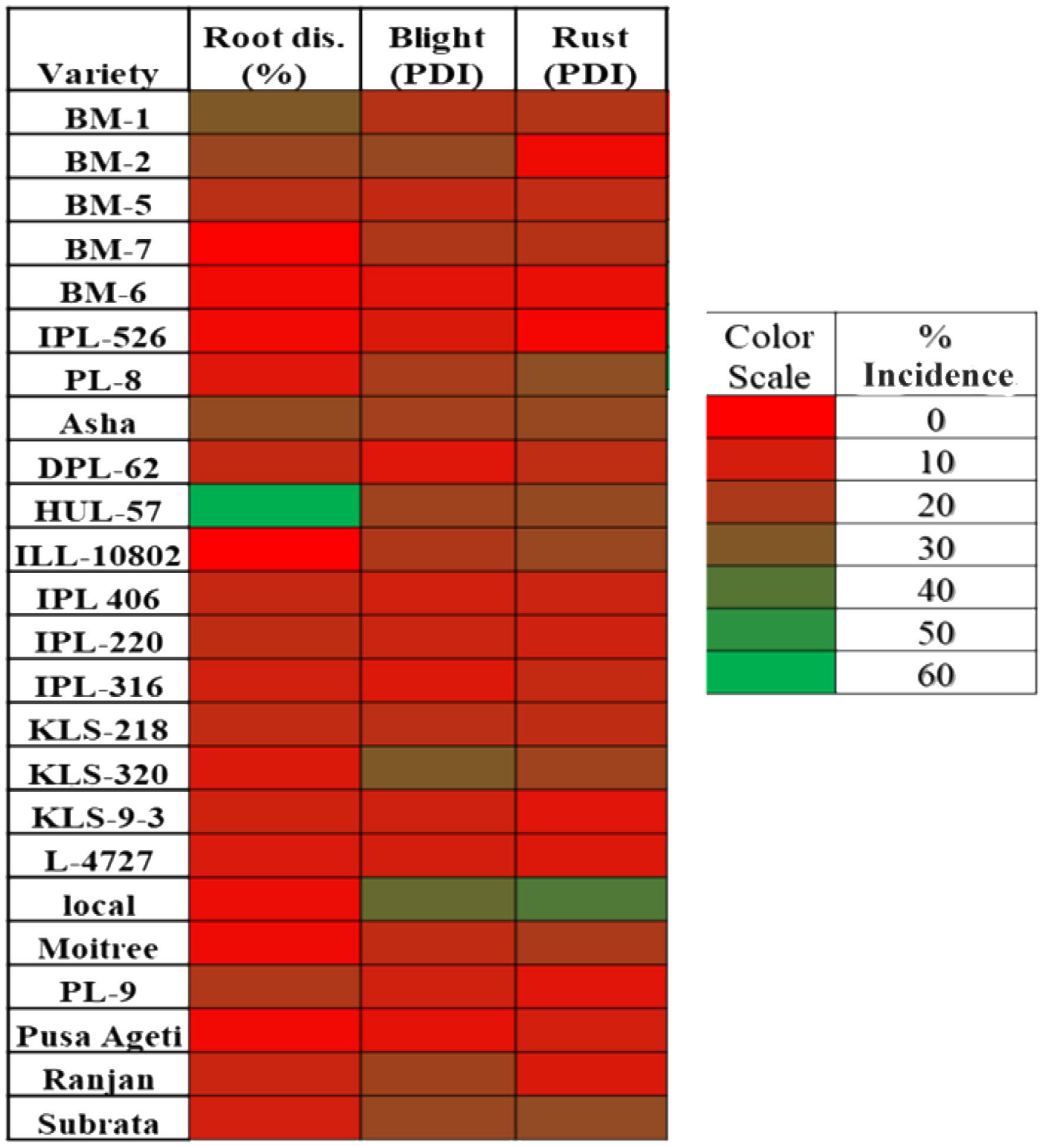
Heat map showing the varietal effect on disease incidence % of collar rot (root disease) and disease severity percent of blight and rust diseases in lentil.

**Figure 5:**
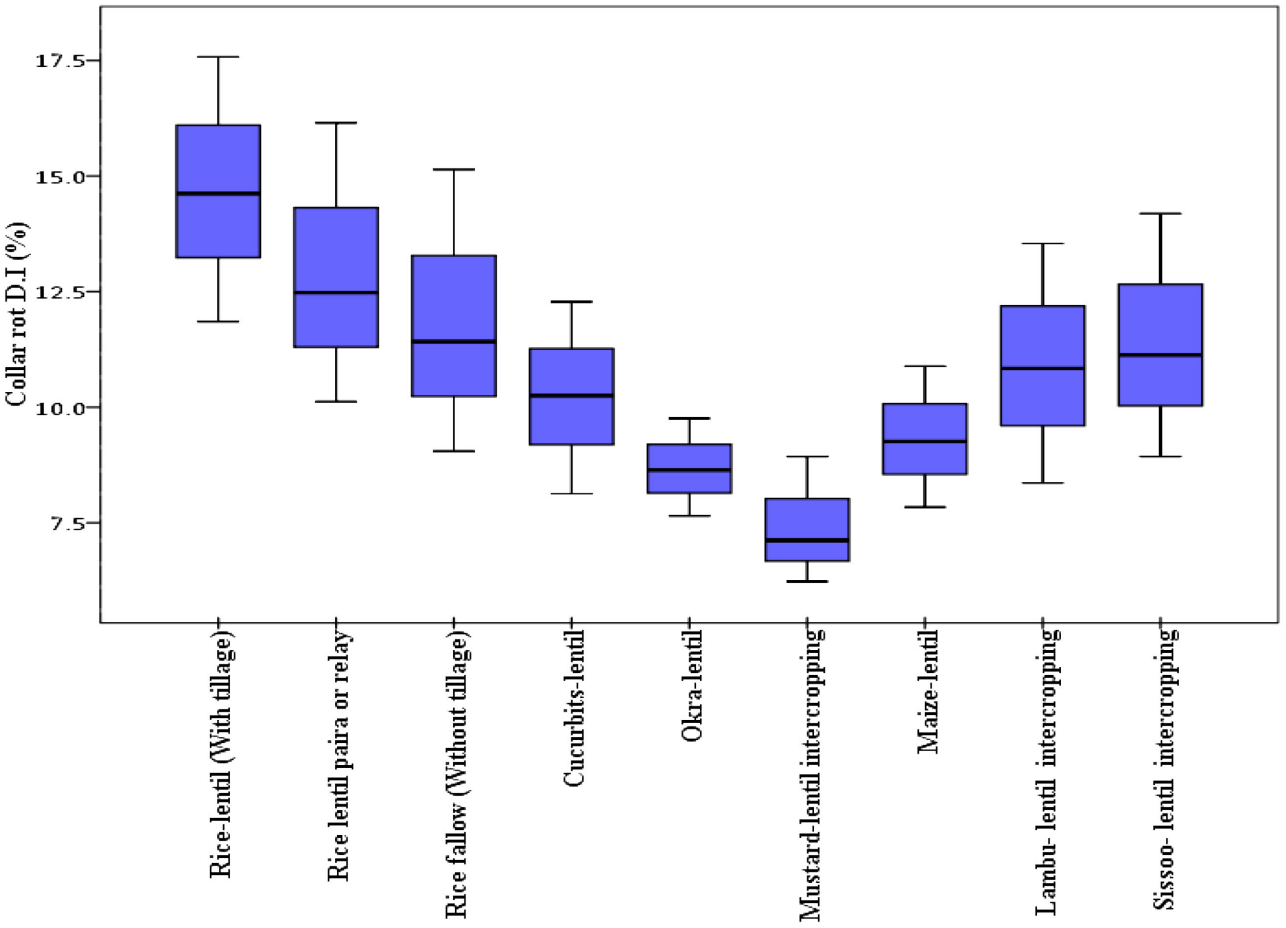
Box plot representing percentage disease incidence of collar rot with different cropping patterns. The horizontal line in the box indicates the median of the data values.

Differential DI and DS were observed in lentil because of varied sowing periods (**Figure 3**), different lentil cultivars (**Figure 4**), and intercropping patterns (**Figure 5**). The DI for collar rot was higher in the normal-sown fields than in the late-sown fields. The DS for LBC and rust was higher in the late-sown crop than in the normal-sown crop. The DI for the collar rot ranged from 4.6% to 10% in the normal-sown crop, with a mean DI of 7.53%. The DI (5.07%) was reduced by 3.4%–6.6% when sowing was delayed. The DS for LBC varied from 9.3% to 13.7% (11.6%) in the normal-sown crop, whereas it ranged from 15.1% to 22.2% (18.1%) with a delay in sowing time. The DS of rust ranged from 10% to 14.6% in the normal-sown crop, with a mean DS of 11.9%. Increase of 17.2%–21.7% in DS (19.4%) for lentil rust was observed with a delay in sowing. Cropping patterns predisposed lentil to a high DI of collar rot, whereas no effect was observed on foliar pathogens. The rice–lentil cropping system had a higher DI than other cropping systems, particularly rice–lentil (with tillage) had the highest DI (14.6%), followed by rice–lentil paira cropping (12.9%) and rice–fallow lentil without tillage (11.8%). Intercropping with mustard (7.4%) and okra (8.6%), individually, considerably reduced the DI for collar rot compared with other intercropping patterns. The varietal effect also influenced the disease pattern in the fields. For collar rot, ILL-10802 was detected as resistant with only 2% DI, and HUL-57 was susceptible with 34.7% DI. For LBC and rust, the highly tolerant cultivars were Pusa Ageti with 5.4% DS and IPL-526 (3.2%), whereas local cultivars (Asha, Ranjan) were susceptible to both diseases.

### 3.3 Cultural and morphological identification of the pathogen

**Figure 6:**
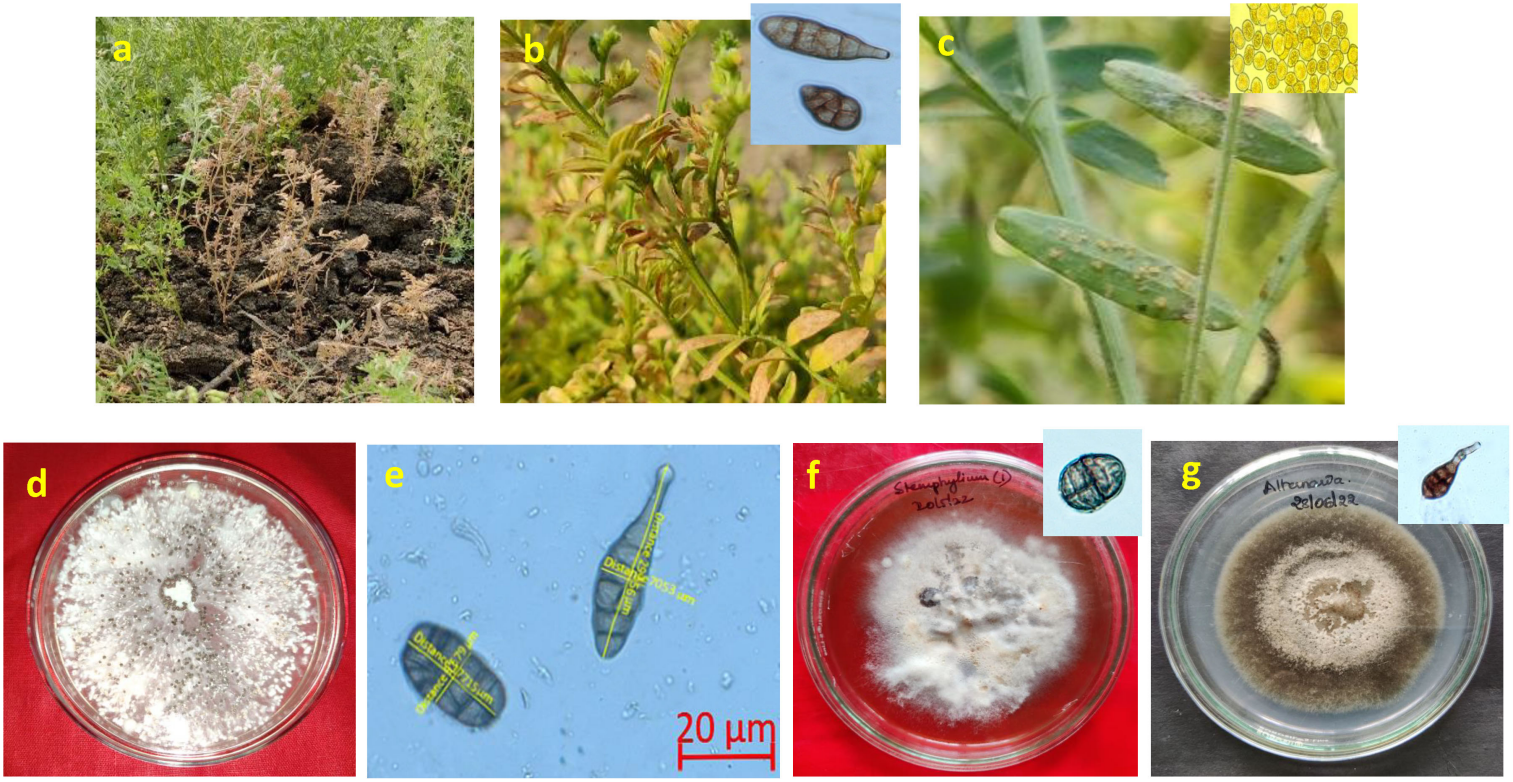
Symptoms of affected lentil plant with disease. **(A)** Collar rot (*Sclerotium rolfsii*) **(B)** Lentil blight complex (*Alternaria/Stemphylium*) **(C)** Lentil rust (*Uromyces viciae fabae*). **(D)** Culture morphology of *Sclerotium rolfsii* causing collar rot disease in lentil. **(E)** Microscopic view of mixed infection of *Alternaria* and *Stemphylium* causing lentil blight complex (LBC) **(F)** Culture morphology and conidia structure of *Stemphylium botryosum* **(G)** Culture morphology and conidia structure of *Alternaria alternata*.

Root pieces infected by collar rot were inoculated on PDA. They had a profusely thick tuft-like white mycelium with centrally arranged, spherical, brown coloured sclerotia (**Figure 6**). For confirmation, its mycelial and sclerotial characteristics were studied, rather than assessing the standard mycological keys as devised earlier (Barnett and Hunter, 1972). For LBC, pathogens were isolated through single spore microscopy of *Alternaria* and *Stemphylium.* Cultures obtained for *Stemphylium botryosum* were velvety to cottony, with dirty whitish brown colonies that were irregular in shape with sector, while those obtained for *A. alternata* were dark green to greyish black in colour and had woolly colonies (**Figure 6**). Microscopic observation revealed the presence of 6–17-µm-long and 5–10-µm-wide conidia in *S. botryosum*. On the contrary, conidia were 17–23-µm long and 2–6-µm-wide in *A. alternata*. Rust uredospore of ellipsoidal shape and 22–29 × 19–23 µm size were recorded from the microscopic observation of the infected rust pustules.

### 3.4 Pathogenicity assay

All cultures obtained from the infected plant areas tested positive for their respective pathogen, *viz*., *Sclerotium rolfsii*, *A. alternata*, and *S. botryosum*. Pathogenicity tests conducted on susceptible cv. HUL-57 for *S. rolfsii* and RKL-22-10 for *A. alternata* and *S. botryosum* confirmed all the corresponding fungal species. Successful establishment of the pathogen revealed that LBC and its causative pathogens, that is, *A. alternata* and *S. botryosum*, were visible 10 days after inoculation, and collar rot occurred within 7 days after inoculation. Based on colony and morphological identification, the pathogens were re-isolated and confirmed as *S. rolfsii*, *A. alternata*, and *S. botryosum*. Thus, Koch’s postulates were completely supported by the re-isolation of all pathogens from the artificially inoculated infected plants.

### 3.5 Molecular characterisation of pathogens

**Figure 7:**
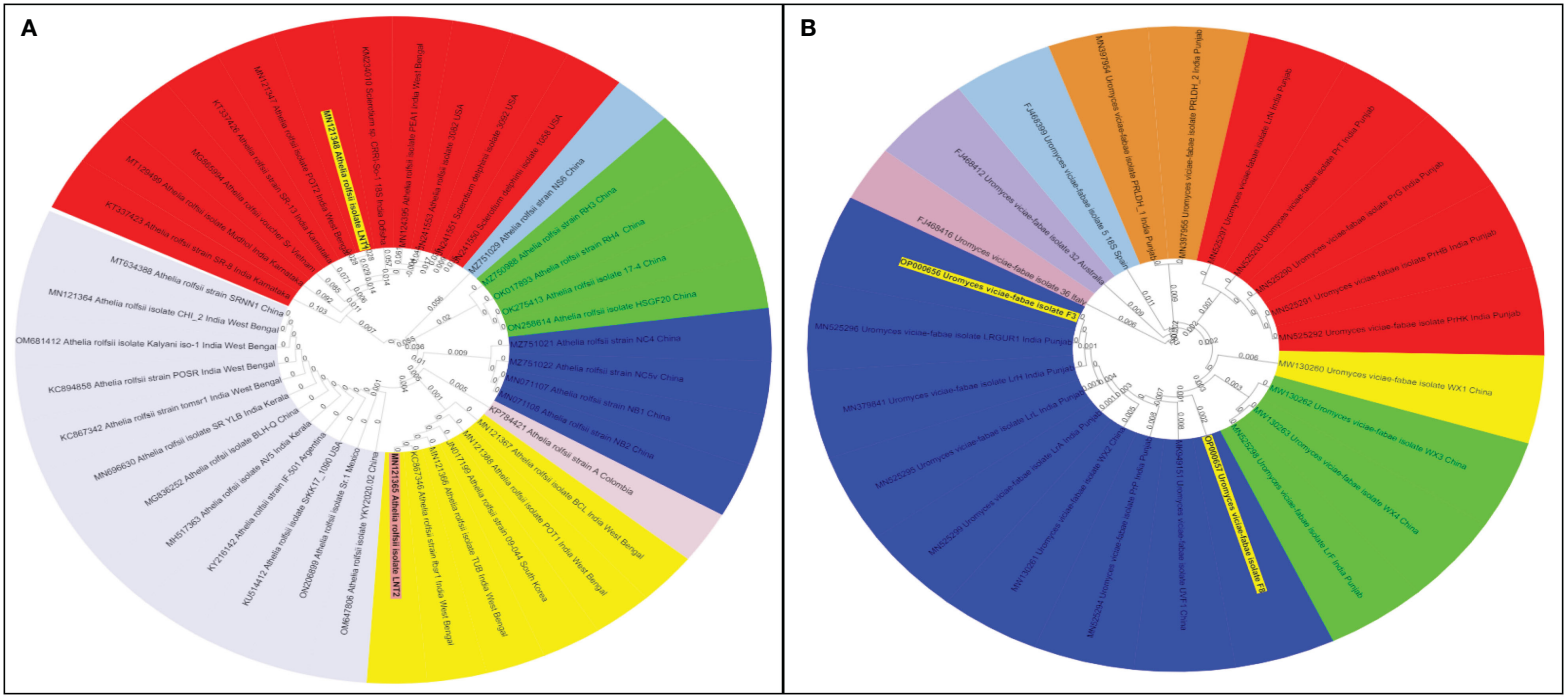
Phylogenetic tree of collar rot and rust pathogens identified based on ITS 18S rDNA gene segments. **(A)** *Sclerotium rolfsii* spp. and **(B)** *Uromyces viciae fabae* spp. The samples from the survey study are indicated by bold text in the tree; all other sequences were obtained from the GenBank database. Different color shades indicate the subgroup of respective pathogen species.

**Figure 8:**
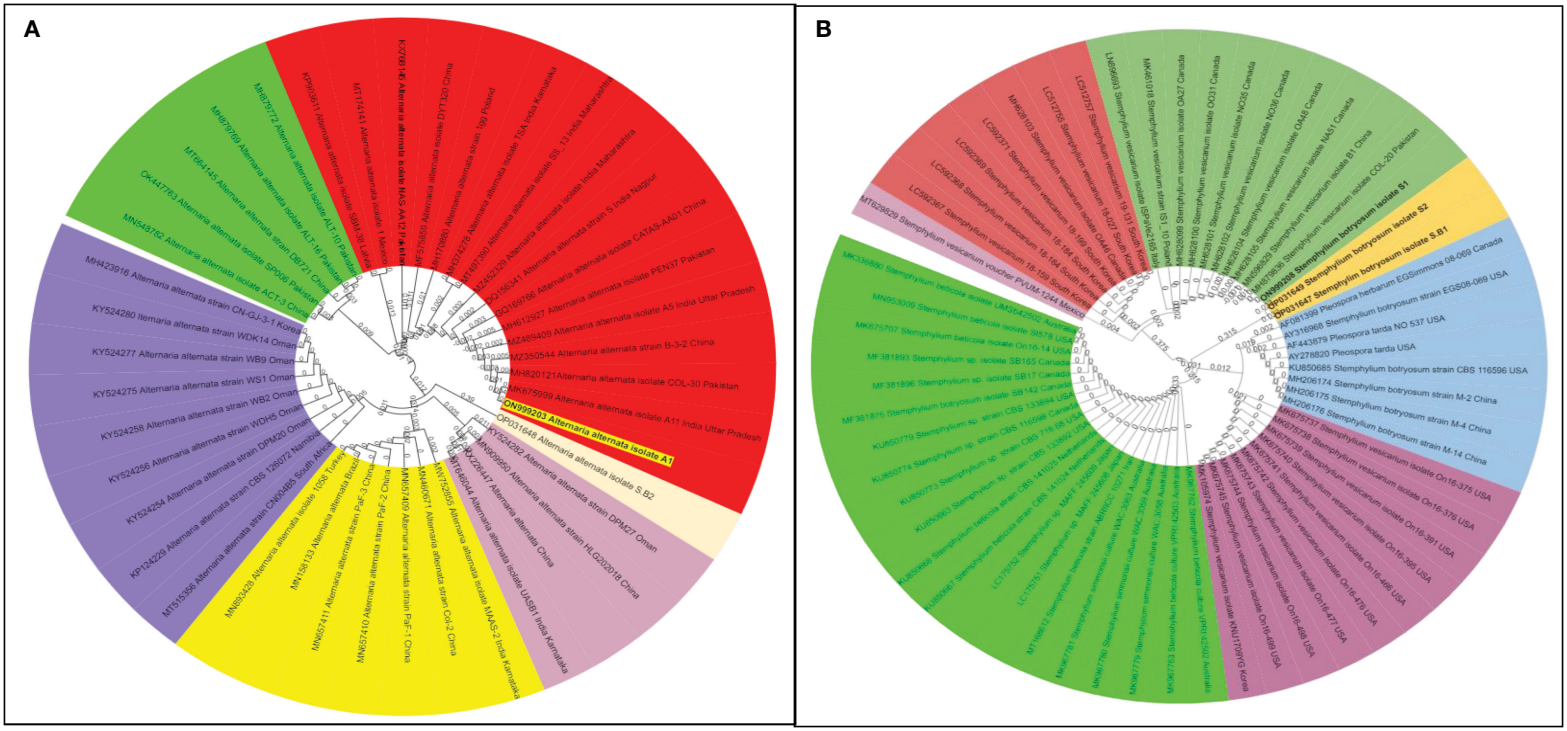
Phylogenetic tree of both the pathogens causing Lentil Blight Complex: *Stemphylium botryosum* and *Alternaria alternata* identified based on ITS 18S rDNA gene segments and glyceraldehyde-3-phosphate dehydrogenase (GAPDH) specific gene segments. **(A)** *Alternaria alternata* spp. and **(B)** *Stemphylium botryosum* spp. The samples from the survey study are indicated by bold text in the tree; all other sequences were obtained from the GenBank database. Different colour shades indicate the subgroup of respective pathogen species.

Like cultural and morphological analyses, positive results were obtained in molecular analysis. The annotations of representative isolates from this investigation with their rDNA sequences submitted to the NCBI GenBank database were as follows: (i) ITS universal primers–accession no. MN121348 LNT1 and MN121365 LNT2 for *S. rolfsii*; ON999203 A1 for *A. alternata*; ON999208 S1 for *S. botryosum*; and OP000656 F3 and OP000657 F8 for *U. fabae* and (ii) GAPDH-specific primers gpd1 and gpd 2–accession no. OP031647 S.B1 and OP031649 S2 for *S. botryosum* and OP031648 S.B2 for *A. alternata* (GPD primers) (**Table 3**). To assess the relationship of the pathogens investigated in this study and their related species, the corresponding genomic regions of 25 related species exhibiting 99%–100% sequence identity with the present isolates were obtained from GenBank, and a phylogenetic tree was constructed. According to the phylogenetic analysis results, the present *S. rolfsii* infecting lentil LNT1 and LNT2 grouped in separate clades, indicating the high genetic variability level among them. In case of rust, F3 and F8 were observed in similar clades, and close resemblance was observed between these isolates and rust isolates from Punjab province of India and China (**Figure 7**). With reference to LBC, *A. alternata* infecting lentil A1 was found to be similar to other *Alternaria* spp., whereas *S. botryosum* S.B2 was highly distinct from other *Alternaria* spp. reported from different hosts from different countries. *S. botryosum* S.B1 and S2 reported in the present study were genetically similar as they were present in the same clade, which indicated that they were different from S1 and other *Stemphylium* spp. reported from different hosts from different countries such as Canada (**Figure 8**).

**Table 3:** Identification and molecular characterization of associated fungi causing lentil diseases in LGB.

Code no.	Primer	Annealing temperature	Amplicon size	Identified as	GenBank accession no.	% Similarity
LNT1	ITS 1 and ITS 4	47°C	700 bp	*Sclerotium rolfsii*	MN121348	100%
LNT2	ITS 1 and ITS 4	47°C	700 bp	*S. rolfsii*	MN121365	100%
A1	ITS 1 and ITS 4	46°C	590 bp	*Alternaria alternata*	ON999203	99.65%
S1	ITS 1 and ITS 4	46°C	600 bp	*Stemphylium botryosum*	ON999208	100%
F3	ITS1 and ITS 4	46°C	640 bp	*Uromyces viciae fabae*	OP000656	100%
F8	ITS1 and ITS 4	46°C	640 bp	*U. viciae fabae*	OP000657	100%
S.B1	gpd1 and 2	55°C	600 bp	*S. botryosum*	OP031647	97%
S2	gpd1 and 2	54°C	600 bp	*S. botryosum*	OP031649	100%
S.B2	gpd1 and 2	55°C	590	*A. alternata*	OP031648	99.80%

### 3.6 Geophytopathological disease distribution mapping

**Figure 9:**
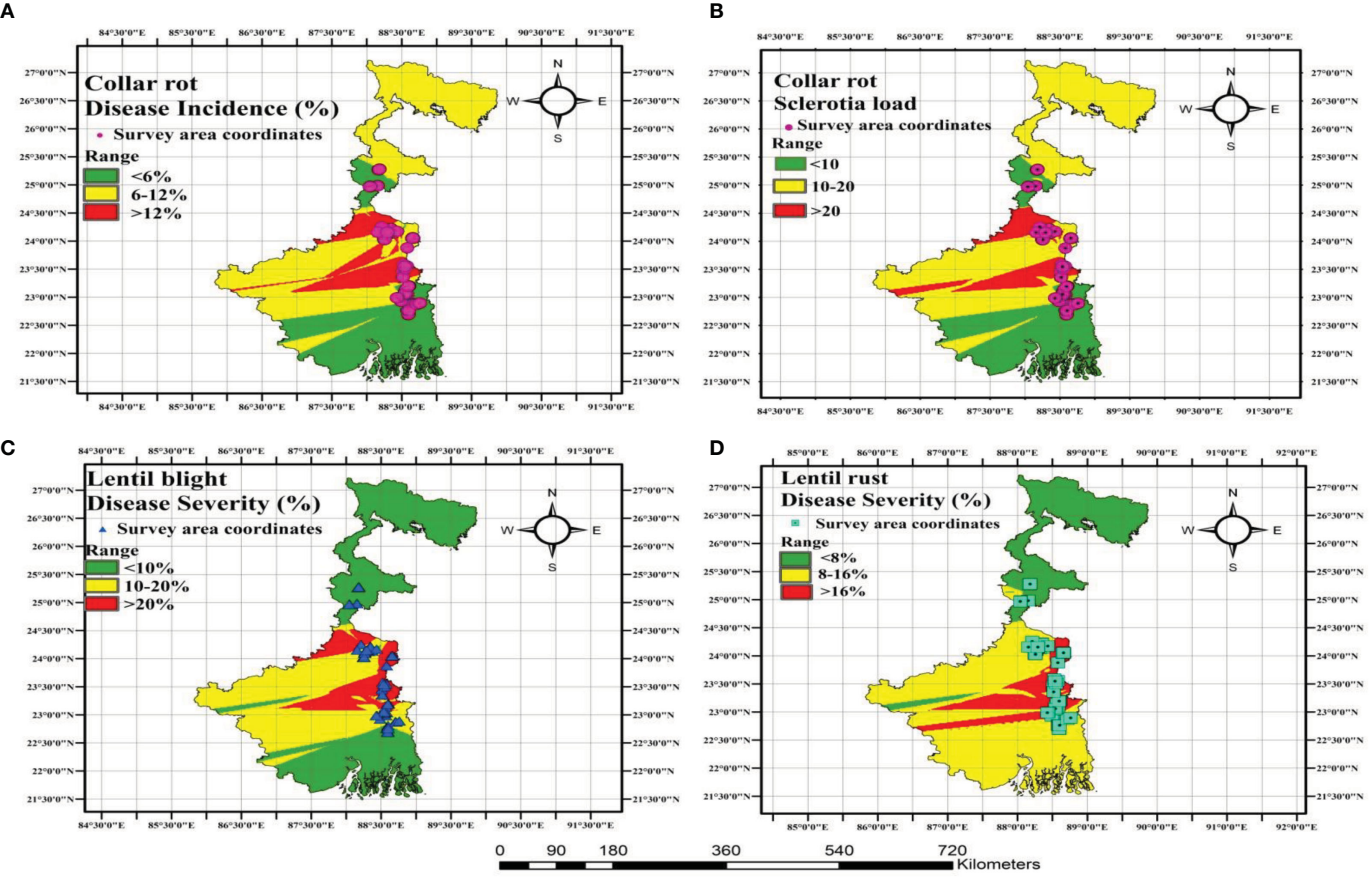
Geophytopathological disease distribution mapping for representing different diseases of lentil growing across major lentil growing areas of LGB. **(A)** Collar rot disease **(B)** Sclerotia load distribution of collar rot disease **(C)** lentil blight complex (LBC) **(D)** Rust disease. Disease demarcation is done through range showing three colours: green colour indicates lower limit, yellow colour medium limit and red colour upper limit.

Spatial disease distribution maps of sample sites were constructed and DI was depicted (**Figure 9**). The highest collar rot percentage was observed in Murshidabad and Nadia, with a disease rating of >12%. The highest percentage of sclerotia load was observed in Murshidabad and Nadia with a >20% range. According to LBC and rust disease severity mapping, the highest DS for LBC and rust was observed in Nadia district and the lowest DS for both the diseases was observed in Malda district. The spatial distribution representation of lentil diseases through mapping was in accordance with the findings of the data obtained through surveying in the farmers’ fields across major lentil-growing areas of LGB.

## 4 Discussion

Lentil is a promising alternative to input and energy-driven cereals in the winter season and can outperform under a low input scenario. This food crop contributes significantly to socio-economic upliftment and nutritional security in India. However, the impact of climate change is prominent in LGB, and due to inclement weather, the incidence of biotic factors has recently increased, as reflected in the present survey. Lentil disease dynamics oscillate because of environmental fluctuations, soil properties, and crop production interventions (Kumar et al., 2013; Singh et al., 2016; Maji et al., 2019). Therefore, establishing a judicious crop production strategy and expeditious crop management practices are pertinent for reducing the risk associated with lentil production loss and to enable its cultivation into a more remunerative enterprise.

Survey of lentil fields in various LGB districts revealed that collar rot and LBC were the most common diseases (high DP). Higher infection levels were observed for collar rot considering the DI, followed by LBC and rust based on DS. Resistant cultivars reported were less accessible to most farmers in this belt owing to the poor seed replacement rate (Chauhan et al., 2016). Additionally, farmers and consumers of LGB prefer small seeded lentil cultivars, which are mostly susceptible to prevalent pathogens. Therefore, implementing resistance breeding programme as well as disseminating resistant cultivars can leverage increased lentil productivity. Growing susceptible cultivars in each season increases disease pressure with more fungal inoculum of both soil and seed-borne diseases. Moreover, most farmers in the surveyed areas mainly depended on locally available lentil cultivars that are collected from equivocal sources, highlighting greater possibilities of disease prevalence.

The survey revealed that changing planting dates is the plausible intervention for reducing the disease risk in lentil. Paradoxical responses were observed for soil-borne and foliar pathogens. Compared with late sowing, the normal date of planting during October to November triggered higher incidence of collar rot in lentil because of erratic rainfall during pre-sowing and availability of excessive soil moisture along with high temperature at the seedling stage, which is in accordance with the previous finding (Sharma et al., 2014). However, late sowing (December) was associated with more foliar disease than normal sowing. The increasing temperature during the flowering stage elicited LBC and rust. LBC occurs usually during the flowering stage (Kumar, 2007). With an elevation in temperature, this disease can also be observed during the pre-flowering stage. Earlier findings have suggested that planting between late October and early November can drastically reduce the potential risks associated with *Stemphylium* blight, which is in line with the findings of the present study (Subedi et al., 2015; Paudel et al., 2020). Lentil rust occurs mostly in January–February in the form of pycnia and aecia. Aeciospores germinate at 17°C–22°C, which is a condition highly congenial for infection if late sowing is performed (Sharma et al., 2014); this result is in close agreement with our results. As lentil is mostly cultivated in the rice-based cropping system in LGB (Yadav et al., 2017; Pramanik et al., 2020), early harvesting of preceding rice through growing short-duration rice cultivars can enhance performance of the rice–lentil system.

Diversifying the cropping system to mitigate pathogen infections is possibly the most seminal near-term strategy towards formulating environment-friendly crop protection schedules. Intercropping can alter the congenial environment for pathogenic invasion as protective biomolecules are secreted with an antagonistic effect and the activity of beneficial microbes is boosted, which altogether leads to association resistance (Hooks and Johnson, 2003; Chadfield et al., 2022). Significant differences were observed in disease incidence among different cropping patterns, which indicates that intercropping influences disease occurrence, especially in case of soil-borne pathogens such as *S. rolfsii*. The present survey detected that lentil intercropped with mustard exhibited maximum reduction in DI of collar rot. This is because Indian mustard (*Brassica juncea* L.) accumulates a high glucosinolate (allyl glucosinolate or sinigrin) level, which produces a large amount of volatile allyl isothiocyanate (AITC) upon hydrolysis. AITC can be successfully employed as a potential biofumigant (Mayton et al., 1996). The present finding corroborated those of earlier studies investigating successful reduction of collar rot of betelvine (Garain et al., 2021) and groundnut (Yella Goud et al., 2013).

Climate changes have exhilarated tremendous changes in the temporal and spatial distribution of several diseases, which were minor in occurrence at one time but now have assumed significance in the lentil production system of LGB. Although collar rot was not a major problem in lentil production during the past years, the augmentation of rice–lentil cultivation, along with erratic rainfall, has increased the incidence of collar rot, thereby gaining the status of a disease of national importance that needs to be monitored and curbed (Iqbal et al., 1995). Collar rot DI and survival and population of sclerotia in a particular soil system are governed by several soil physiochemical factors such as soil texture. As majority of the districts surveyed in our study belong to the new alluvial zone, which has sandy clay loam soil, collar rot DI was higher in this zone than in the old alluvial zone with mostly clayey soil. According to Punja (1985), lighter soils are more conducive to collar rot. A previous study confirmed the maximum incidence (100%) of collar rot in sandy clay loam and minimum incidence (43.33%) in clay loam (Banyal et al., 2008; Mahato et al., 2017), thereby supporting the present study findings. Light soils have better porosity and aeration and can retain comparatively less moisture. Sandy clay loam soil can most significantly fulfil the high oxygen demand for sclerotia survival, thus favouring better growth and spread of the pathogen compared with heavy textured soils.

Climate change has a strong impact on blight incidence in lentil. Once *Stemphylium* blight was a minor disease with local significance; however, it has become a major threat after its first detection in Bangladesh and adjacent LGB in 1986 (Das et al., 2019). The emergence of *Alternaria* blight has also been recently associated with yield loss risk in lentil production in LGB (Roy et al., 2021). The present survey characterised the occurrence of mixed infection of *Stemphylium* and *Alternaria* in several instances. As *Alternaria* and *Stemphylium* are more closely related, they form a pathogen complex and produce symptoms in the infected leaves of lentil that are almost indistinguishable with only a minor difference. The blight spots were angular in *S. botryosum* (Mandal et al., 2019) but concentric in *A. alternata.* Researchers and lentil growers find pathogen detection and subsequent disease management challenging when lentil develops a mixed infection of both blight-causing pathogens. Additionally, difficulty in sporulation and conidia production under laboratory conditions, especially of *S. botryosum*, imposes a great affliction for intervening blight risk. Moreover, previous findings were limited to the screening of lentil genotypes against *S. botryosum* (Podder et al., 2013; Das et al., 2017; Razzak et al., 2018) and *A. alternata* (Roy et al., 2021). To the best of our knowledge, this is the first report of successful isolation of *S. botryosum* in India and further confirmation of the pathogen through sequencing by using ITS and *Stemphylium*-specific gpd1 and gpd2 primers. Therefore, the present report affirmed the occurrence of a complex form of blight in lentil due to mixed infection of both pathogens *Alternaria* and *Stemphylium*. Further studies are warranted to unveil the interaction between both these pathogens to confirm their synergistic or antagonistic relationship for prudent crop management to mitigate the lentil blight bottleneck.

The present study highlighted that collar rot, LBC, and rust are daunting barriers for successful lentil cultivation in LGB. No individual occurrence of *Alternaria* or *Stemphylium* blight was observed, rather a mixed infection was eventuated in a complex manner. The symptoms of LBC are confusing and could be explicitly ascertained by integrating spore morphology through microscopy and molecular techniques. *Stemphylium-* and *Alternaria*-specific primers such as gpd1 and gpd2 confirmed the presence of both pathogens. Planting of lentil during the second fortnight of November can ameliorate the climate-mediated disease risk. Soil texture plays a significant role in collar rot development, and this is more pronounced in light textured soil. Additionally, evaluation and identification of resistant genotypes against multiple diseases, development of associated resistance through intercropping with mustard, accurate diagnosis, and accurate pathogen detection can effectively break the pathogen cycle and subsequently curtail the disease threat, thereby encouraging the adoption of successful lentil cultivation in this zone.

## Data availability statement

The datasets presented in this study can be found in online repositories. The names of the repository/repositories and accession number(s) can be found in the article/**Supplementary Material**.

## Author contributions

HN: Writing – original draft, preparation, Investigation, carried out the experiment. SB, HN: Formal analysis, phylogenetic analysis. SD, AD, RD: Validation. RD, AD, SD, SP, SR: Resources, Disease survey. AD, SD, RD: Investigation, Conceptualization, Writing, review & editing. SG and SK: Review & Supervision, Resource. All authors contributed to the article and approved the submitted version.
