# Supplementary material for: Disease spectrum and its molecular characterisation in the lentil production system of lower-Indo Gangetic plains
Source: Front Plant Sci. 2024 Feb 23;15:1199016. doi: 10.3389/fpls.2024.1199016 (PMC10920269; doi:10.3389/fpls.2024.1199016)
Supplement: Supplementary file 1 [file Table_1.docx]

**Table S1: Characteristics of lentil fields surveyed across the districts of LGB during three years (2018–2021)**

|  | | **Number of fields surveyed** | | | **Number of fields infected** | | | | | | | | | **Geographical coordinates** | | **Soil texture** | **Cropping sequence** |
| --- | --- | --- | --- | --- | --- | --- | --- | --- | --- | --- | --- | --- | --- | --- | --- | --- | --- |
| LGB  Districts | Blocks | 2018  -19 | 2019-20 | 2020-21 | **Collar rot** | | | **Blight complex** | | | **Rust** | | | **Latitudes** | **Longitudes** |  |  |
|  |  |  |  |  | 2018  -19 | 2019  -20 | 2020  -21 | 2018-19 | 2019  -20 | 2020  -21 | 2018  -19 | 2019  -20 | 2020  -21 |  |  |  |  |
| Nadia | Chakdaha | 6 | 13 | 8 | 6 | 12 | 7 | 5 | 13 | 8 | 5 | 13 | 8 | 23.0297°N | 88.5377°E | Sandy clay loam | Rice fallow lentil  Rice-lentil paira  Rice-lentil (with tillage) |
|  | Ranaghat | 6 | 4 | 4 | 5 | 2 | 4 | 5 | 4 | 4 | 5 | 4 | 4 | 23.1926°N | 88.5956°E |  |  |
|  | C-Block (Kalyani) | 6 | 4 | 4 | 5 | 4 | 3 | 5 | 4 | 4 | 0 | 4 | 4 | 22.9882°N | 88.4280°E |  |  |
|  | Krishnagar-I | 6 | 4 | 5 | 6 | 4 | 5 | 6 | 4 | 5 | 5 | 4 | 5 | 23.3553°N | 88.5147°E |  | Mustard-Lentil intercropping  Rice-lentil (with tillage)  Maize-Lentil |
|  | Chapra | 6 | 6 | 4 | 5 | 5 | 4 | 6 | 6 | 4 | 6 | 6 | 3 | 23.5080°N | 88.5541°E |  | Rice-lentil paira  Vegetables-Lentil |
|  | Karimpur | 8 | 4 | 4 | 7 | 3 | 4 | 8 | 4 | 4 | 7 | 4 | 3 | 24.0584°N | 88.6568°E |  | Jute-Lentil-Moong  Vegetables-Lentil |
|  | **Total** | **38** | **35** | **29** | **34** | **30** | **27** | **35** | **35** | **29** | **28** | **35** | **27** |  | | | |
| Murshidabad | Sagardighi Block | 8 | 17 | 19 | 7 | 14 | 19 | 7 | 16 | 19 | 3 | 15 | 18 | 24.1002°N | 88.2667°E | Clay loam | Rice-lentil (With tillage)  Rice-fallow (Without tillage) |
|  | Murshidabad-Jiaganj (MJ Block) | 4 | 11 | 5 | 4 | 8 | 5 | 3 | 11 | 5 | 3 | 8 | 5 | 24.2176°N | 88.3498°E | Sandy clay loam | Rice-lentil (With tillage)  Rice-lentil paira |
|  | Berhampore | 4 | 4 | 4 | 4 | 4 | 4 | 4 | 4 | 4 | 4 | 4 | 4 | 24.2543°N | 88.2102°E |  |  |
|  | **Total** | **16** | **32** | **28** | **15** | **26** | **28** | **14** | **31** | **28** | **10** | **27** | **27** |  | | | |
| Malda | English Bazar | 6 | 4 | 4 | 5 | 3 | 4 | 4 | 4 | 4 | 0 | 3 | 3 | 24.9835°N | 88.1553°E | Clay | Rice-lentil (With tillage) |
|  | Kaliachak-2 | 4 | 4 | 4 | 4 | 4 | 3 | 4 | 4 | 4 | 4 | 3 | 4 | 24.9667°N | 88.0459°E |  | Rice-lentil (With tillage)  Rice-lentil paira |
|  | Gazal block | 5 | 6 | 9 | 4 | 5 | 9 | 4 | 5 | 9 | 4 | 6 | 9 | 25.2705°N | 88.1722°E | Clay loam | Rice-lentil (With tillage) |
|  | **Total** | **15** | **14** | **17** | **13** | **12** | **16** | **12** | **13** | **17** | **8** | **12** | **16** |  | | | |
| North 24 Parganas | Habra -1 | 4 | 4 | 4 | 3 | 2 | 4 | 4 | 4 | 4 | 3 | 4 | 4 | 22.8743°N | 88.7138°E | Clay loam | Vegetables-lentil  Rice-lentil (With tillage) |
|  | Barasat block-1 | 4 | 4 | 4 | 4 | 4 | 3 | 4 | 3 | 4 | 4 | 3 | 4 | 22.7594°N | 88.5832°E | Sandy clay loam | Rice-lentil (With tillage)  Vegetables-lentil |
|  | Barasat block-2 | 4 | 8 | 7 | 4 | 6 | 7 | 3 | 8 | 7 | 3 | 8 | 4 | 22.6951°N | 88.5959°E | Clay loam | Rice-lentil (With tillage)  Vegetables-lentil |
|  | **Total** | **12** | **16** | **15** | **11** | **12** | **14** | **11** | **15** | **15** | **10** | **15** | **12** |  | | | |
| **Grand Total (267)** | | **81** | **97** | **89** | **73** | **80** | **85** | **72** | **94** | **89** | **56** | **89** | **82** |  |  |  |  |
